# During a short window of Drosophila oogenesis, piRNA biogenesis may be boosted and mobilization of transposable elements allowed

**DOI:** 10.3389/fgene.2014.00385

**Published:** 2014-11-06

**Authors:** Jérémy Dufourt, Chantal Vaury

**Affiliations:** ^1^Centre National de la Recherche Scientifique, Unité Mixte de Recherche6293, Institut National en Santé et Recherche Médicale, Unité Mixte de Recherche1103Clermont Ferrand, France; ^2^Laboratoire GReD, Clermont Université, Université d'AuvergneClermont Ferrand, France

**Keywords:** transposable elements, piRNA, Piwi, evolution, spatio-temporal regulation

## Introduction

In *Drosophila*, PIWI-interacting RNAs (piRNAs) form a distinct class of small 24–30 nt single-stranded non-coding RNAs that are associated with the PIWI proteins Piwi, Aubergine (Aub), and Argonaute 3 (AGO3), which are expressed in the germline (Brennecke et al., 2007). Genetic studies have shown that the PIWI/piRNA ribonucleoproteic (RNP) complex has an evolutionary conserved role in the silencing of transposable elements (TEs) in the germline of animals ranging from sponges to humans (Cerutti and Casas-Mollano, 2006; Aravin et al., 2007; Grimson et al., 2008; Luteijn and Ketting, 2013).

piRNAs arise from specific genomic clusters called piRNA clusters that are mainly localized in pericentomeric regions. These clusters are transcribed uni/bi-directionnally as long single-stranded RNA precursors by the RNA polII (Sienski et al., 2012; Goriaux et al., 2014; Mohn et al., 2014). In the germline, these precursors are processed into piRNAs within specific cytoplasmic regions called the nuage prior to being loaded onto Piwi and Aub proteins. The piRNAs generated from this initial transcription of piRNA clusters are called primary piRNAs. These RNP complexes can repress expression of TEs at the transcriptional (TGS) (Sienski et al., 2012; Rozhkov et al., 2013) and/or post-transcriptional (PTGS) level (Chambeyron et al., 2008; Haase et al., 2010; Dufourt et al., 2011; Rozhkov et al., 2013).

TGS control results from a compact repressive chromatin structure characterized by an enrichment of histone H3, which is tri-methylated on Lysine 9, the so-called H3K9me3 repressive mark. It has been suggested that Piwi loaded onto piRNAs identifies TE targets by a homology-dependent base-recognition mechanism. It recruits SU(Var)3-9, the major heterochromatin-specific HMTase in *Drosophila* (Huang et al., 2013), which tri-methylates histone H3 on Lysine 9 (H3K9me3) of the target. Several studies have reported that this heterochromatic structure is labile (Dufourt et al., 2011; Sienski et al., 2012), indicating that TEs can occasionally bypass repression (Haase et al., 2010; Rozhkov et al., 2013; Klenov et al., 2014).

piRNAs also serve as guides for post-transcriptional repression (PTGS) of TEs. PTGS involves two other PIWI proteins, Aub, and Ago3. The Aub/piRNA RNP complex targets cytoplasmic mRNAs encoded from TEs and cleaves the mRNA by virtue of its slicer activity. This gives rise to new piRNAs called secondary piRNAs, which are sense with respect to the canonical transposon mRNAs. These secondary piRNAs are loaded onto Ago3. The Ago3/piRNA RNP complex targets precursor transcripts arising from piRNA clusters. Subsequent cleavage prompts the biogenesis of new piRNAs bound to Aub whose sequence is identical to that of the initiator piRNA. This loop of amplification, called the ping-pong cycle, amplifies silencing-competent piRNAs (Brennecke et al., 2007).

Acting together, these two mechanisms, TGS and PTGS, tightly repress TE transposition in the germ line and thus act as guardians of genome integrity. Interestingly, since the pool of piRNAs produced in the oocyte is deposited in the embryo, TE repression is transmitted from the mother to her progeny, who are immediately protected against TE mobilization. (Ronsseray et al., 1993; Malone et al., 2009; Handler et al., 2011; de Vanssay et al., 2012; Grentzinger et al., 2012; Le Thomas et al., 2014).

Since TE transcription is blocked by TGS in the germ line, the current model fails to define when the ping-pong cycle is active and if a reboostrap has to occur to increase the stock at each generation. It also fails to explain why, despite this tight repression, high genetic variability, mainly due to TE insertions, is observed in each genome sequenced to date, evidence suggesting that TEs have a means of overcoming repression in the germ line and transpose.

In the germarium, at the anterior side of Drosophila ovaries, the germline stem cell (GSC) divides to give a daughter cell called the cystoblast. This latter undergoes four cycles of mitotic division to form cysts of successively 2, 4, 8, and 16 germ cells. The mature egg chamber consisting of 16 germ cells including the oocyte and 15 nurse cells then leaves the germarium. Recently, we observed that transient downregulation of Piwi occurs during early oogenesis when cysts divide (Dufourt et al., 2014). In this region called the Piwiless pocket (Pilp), the absence of Piwi is correlated with a decrease in germline repression exerted on sensor transgenes used as read-out of repression of two TEs, the LTR retrotransposon Idefix and the P-transposon. We propose that this short window of oogenesis could correspond to the moment at which mRNAs are synthesized from TEs, as a consequence of which the ping-pong cycle is boosted, resulting in an increasing pool of piRNAs, and TE replication cycles are allowed.

### Enhancing the pool of germline piRNAs

It has been shown that ping-pong processing ensures that a large pool of piRNAs will be produced during oogenesis and transmitted to protect the embryo as soon as it starts developing. Because of the TGS exerted on TEs in the germ line, the moment at which this increase occurs remains unknown. It could be speculated that the pool of piRNAs is increased within the primordial germ cells (PGC), where maternally-deposited piRNAs are present and transcription of piRNA clusters is active (Le Thomas et al., 2014). However, the repressive heterochromatin structure embeding TEs would be expected to prevent the production of TE mRNAs and thereafter any new round of secondary piRNA synthesis from TE mRNAs.

**Figure 1:**
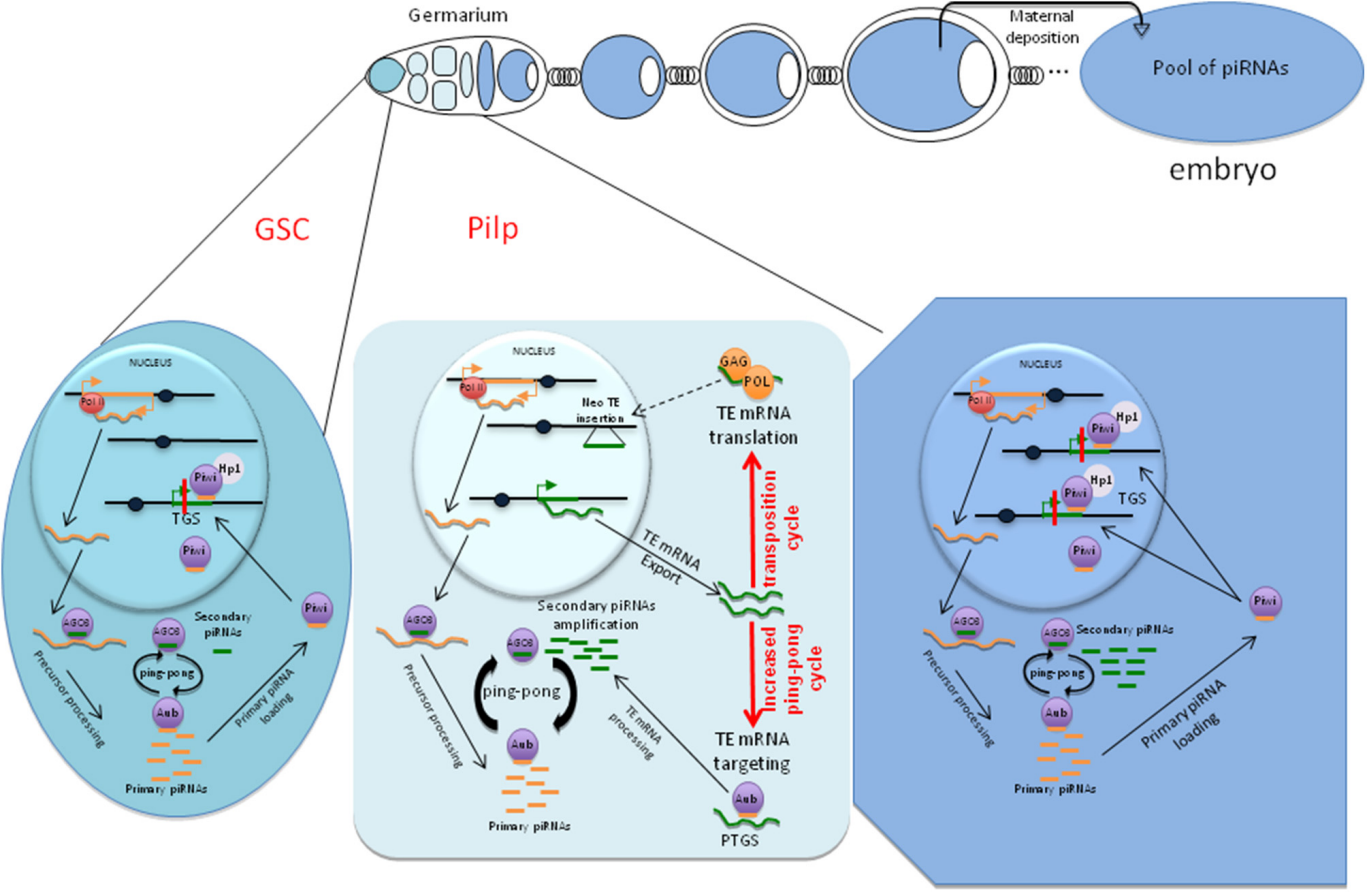
**A schematic representation of *Drosophila melanogaster* egg chambers**. In the germarium (left part of the upper panel), the Pilp is shown as light blue cells. Transcriptional gene silencing (TGS) and post-transcriptional gene silencing (PTGS) targeting TEs are presented below. In the Pilp (mid cell, lower panel), the decrease in Piwi allows TE transcription, which has two consequences on the TE/host relationship (red arrows): (1) an increased pool of piRNAs produced by the ping-pong mechanism and (2) increased transposition cycles leading to neo-TE insertions.

We propose that, in the Pilp, the decrease in Piwi diminishes the TGS exerted on TEs and leads to their transcription. The resulting mRNAs serve as targets for the ping-pong cycle, which is thus kicked-up and the piRNA pool that will be ultimately transmitted to the progeny is amplified. This phase is transient and restricted to the dividing cysts because Piwi expression is restored to normal at the end of the mitotic divisions (Figure 1).

Interestingly, we recently observed that Aub, a major component of PTGS, is required for TE silencing during the germarial stages of oogenesis whereas its depletion after this stage has no impact on TE silencing (Dufourt et al., 2014). In contrast, Piwi involved in TGS is required throughout oogenesis. Together with the existence of the Pilp, where TGS is weakened, these data suggest that the PTGS exerted on TEs mostly takes place in the small group of cells where TE mRNAs are produced.

Altogether, these results designate dividing cysts and more specifically the Pilp as a window of the germ line development during which mRNAs encoded by TEs may be produced, the ping-pong cycle boosted and the pool of *de novo* piRNAs that will be inherited amplified.

### Allowing TEs to transpose in the germ line

Although they represent a constant threat for genome stability, TEs have efficiently colonized all the eukaryotic genomes and are considered as major tools for genome evolution and plasticity. This implies that they find a way to bypass host defense mechanisms and mobilize in the germ cells thereby ensuring their propagation to the next generation.

We believe that the best moment when TEs can escape piRNA silencing and insert the genome is when cysts divide, thus within the Pilp. Three lines of evidence support this assumption. First, loss of control of TEs in the Pilp will not affect the potential of the stem cell to continuously produce new viable germline cysts. Mobilization in the GSC would guarantee new insertions to the whole progeny but could also create severe genome damage that might lead to loss of GSC and sterility or lethal effects on descendants. Thus, protecting the stem cell from which all the future germ cells will derive appears to be essential for the species. Second, in the Pilp, the oocyte is not yet in a condensed state that could prevent TE activity. The oocyte nucleus will be condensed and blocked in meiosis after the cystoblast has completed the four rounds of mitotic division to create a cyst. Third, TE silencing is weakened owing to a decrease in Piwi. Transcription is then allowed and replication cycles may start from the pool of synthesized mRNAs.

Because of its property to display a weakened RNA silencing, the Pilp may thus ensure the constant and controlled permissiveness for *de novo* TE genomic integrations (Figure 1) and also sporadic bursts of TE transpositions, as occasionally mentioned in the literature (Biemont and Vieira, 2006). It is noteworthy that TE activation has been reported in the germline of several species(Zamudio and Bourc'his, 2010). In *Arabidopsis*, the maternal endosperm genome is hypomethylated, resulting in transient transposon activation (Hsieh et al., 2009). In pollen from *Arabidopsis*, TEs are also reactivated and transpose but only in the pollen vegetative nucleus thereby avoiding dramatic events in the sperm cells, which give rise to the progeny (Slotkin et al., 2009). In mice, genome-wide loss of DNA methylation accompanies the acquisition of pluripotent states in PGC, which opens a window of opportunity for TEs to escape from host restraint (Rougier et al., 1998; Hajkova et al., 2002). L1 transcripts and proteins are found in male germ cells entering meiosis but are repressed in differentiated somatic tissues (Branciforte and Martin, 1994). As a last example, the MT family of LTR retrotransposons, although it represents only <5% of the genome, accounts for 13% of the transcriptome of the mature mouse oocyte (Peaston et al., 2004). In all these examples, TE activation is not only linked to the availability of key transcription factors but also to a relaxation of epigenetic control in the cells.

## Perspective

The two potential functions of the Pilp, i.e., increasing the pool of piRNAs and allowing TE transposition, are shown in Figure 1.

To have a better understanding of the close relationship between TE, evolution, germline protection and transmission to the progeny, it will be important to show whether the piRNA pool in the Pilp is different from that in the GSC and/or the rest of the ovaries. It would be expected for piRNAs arising from piRNA clusters to be highly abundant in GSC but unable to complete the ping-pong cycle because of TGS repressing TE transcription. In contrast, piRNAs transmitted to the Pilp by the GSC should be able to complete the ping-pong cycle with piRNA partners arising from TE mRNAs. To have a fuller understanding of this process, studies should be made to investigate whether these mechanisms are conserved across species to maintain a harmonious balance between TEs and their host genome.

Several studies have recently shown the involvement of the piRNA pathway in additional functions such as germ line development and sex determination (Rouget et al., 2010) (Kiuchi et al., 2014). We also reported that the downregulation of Aub after the germarium stage leads to sterility whereas, at the same stage, TEs are repressed (Dufourt et al., 2014). A recent study demonstrated that Piwi/Su(Var)3-7 genetic interaction leads to an increase in sterility and embryo defects but, importantly, does not correlate with TE derepression (Basquin et al., 2014). Hence, it will be important to explore the role of this short window of germ line development not only in TE control but also in total mRNA regulation and germ line development.

### Conflict of interest statement

The authors declare that the research was conducted in the absence of any commercial or financial relationships that could be construed as a potential conflict of interest.
